# Atypical Presentation of Babesiosis With Neurological Manifestations as Well as Hematological Manifestations

**DOI:** 10.7759/cureus.26811

**Published:** 2022-07-13

**Authors:** Tejaswi Venigalla, Christine Adekayode, Shriya Doreswamy, Hussein Al-Sudani, Supriya Sekhar

**Affiliations:** 1 Internal Medicine, Einstein Medical Center Montgomery, East Norriton, USA

**Keywords:** tick borne infections, lyme's disease, parasites, malaria, hemolytic anemia, altered mental status, tick bite, stroke-like, neurological manifestations, babesiosis

## Abstract

Babesiosis is an infectious disease that is typically known to present with fevers, chills, and myalgias; and less commonly with anorexia, headache, nausea, and vomiting. The least common are shortness of breath, sore throat, neck stiffness, emotional lability, photophobia, and dark urine. Even more unusual are severe neurologic manifestations like altered mental status, motor deficits, and ataxia.

We present two cases of patients, both in their seventies, with multiple comorbidities, who were admitted with similar symptoms of confusion/cognitive impairment, slurred speech, ataxia, fever, myalgias and chills, urinary frequency, and urgency, with no previous history of travel outside the country or tick bites. Both patients had extensive workup, which raised suspicion of hemolytic infections, especially babesiosis and malaria. Considering our patients had not traveled out of the country, we leaned more toward babesiosis. The patients were treated appropriately for babesiosis and were also empirically treated for Lyme's, anaplasmosis, along with Mycoplasma in the second patient. Following two days of treatment, cognition, as well as speech, improved dramatically. On outpatient follow-up, both patients had entirely resolved hemolysis, parasitic load, and neurological manifestations.

During the literature review, neurologic manifestations, being associated with babesiosis, were found to be exceedingly rare but could be fatal if left undiagnosed. It is an infection that is associated with complete recovery on prompt diagnosis and treatment. It is pertinent to have a high suspicion of this disease, especially in endemic areas, such as the Northeast United States, even more so when seen with hematologic and neurologic manifestations.

## Introduction

Babesiosis is an infectious disease caused by a protozoon of the genus Babesia, which infects and lyses red blood cells. Babesiosis is typically known to present as fevers, chills, and myalgias; and less commonly with anorexia, headache, nausea, and vomiting. The least common are shortness of breath, sore throat, neck stiffness, emotional lability, photophobia, and dark urine. Even more unusual are severe neurologic manifestations like altered mental status, motor deficits, and ataxia [1-6]. So, when we see neurologic manifestations, it gives a reason to also think about tickborne diseases like babesiosis and Lyme's or anaplasmosis, especially in endemic areas of the Northeastern United States.

We present two cases of patients who were admitted with similar symptoms of confusion, slurred speech, ataxia, fever, myalgias and chills, urinary urgency and frequency, with no previous history of travel outside of the country. They were found to have babesiosis, along with hemolytic anemia. Surprisingly, both patients had incidental hepatitis C antibody positivity. They were treated appropriately for babesiosis, and the first patient was also empirically treated for Lyme’s and anaplasmosis, and the second patient was empirically treated for Lyme's, anaplasmosis, and Mycoplasma. Following two days of treatment, their cognition, as well as speech, improved dramatically. On outpatient follow-up, both patients had entirely resolved hemolysis, neurological symptoms, and lower parasitic load.

## Case presentation

Case #1

A 72-year-old male, a gardener by profession, presented to the hospital with confusion, slurred speech, fever, and urinary urgency and frequency. The patient had nine days of cognitive impairment, slurred speech, and fevers. This neurologic impairment has resulted in a motor vehicle accident. His medical history is significant for sarcoidosis, Reiter's syndrome, rheumatoid arthritis on golimumab infusions, and lymphomatoid papulosis. His only travel history was to Florida, and he has not been outside of the country in the last few years. He denied having any recall of tick bites. He was febrile with a T-max of 39.5 Celsius on admission. His heart rate was 92 bpm, respiratory rate was 16 BR/min, and blood pressure was 141/65 mmHg.

Investigations

A comprehensive metabolic panel (CMP) showed a sodium of 132, elevated blood urea nitrogen (BUN), creatinine of 1.57 (unknown baseline creatinine), elevated total bilirubin of 2.1 and direct bilirubin of 1.1, and alanine transaminase (ALT) and aspartate transaminase (AST) of 127 and 210, respectively. He had no known kidney disease. The complete blood count (CBC) showed hemoglobin and hematocrit of 12.7 and 37.3 with a high red blood cell distribution width (RDW) of 16.7 and a low normal mean corpuscular volume (MCV) of 80.7. He had no leukocytosis and his platelets were 43. Reticulocyte count was 2.3 and immature reticulocyte fraction (IRF) was 16.7. Urinalysis and culture were negative.

His Lyme's titers were negative. Anaplasma phagocytophilum DNA polymerase chain reaction (PCR) was negative. CT head was negative for acute hemorrhage, mass effect, or infarction.

Peripheral Smear

**Figure 1:**
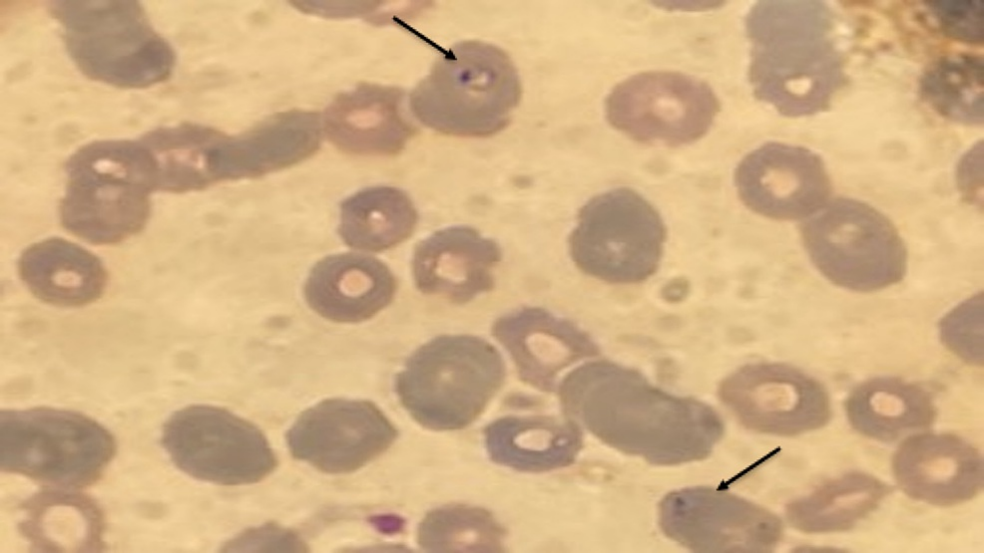
With arrows toward the intracellular ring-like parasites from the patient in Case #1

Peripheral smear showed parasites and a parasitic load of 1.8. It also showed the classic presentation of babesiosis with intracellular ring-like parasites on the peripheral smear (Figure 1).

Differential Diagnosis

The differentials were broad, including babesiosis, malaria, Anaplasma, and Lyme's disease in this patient. Due to the peripheral smear showing ringed erythrocytes and parasites, we had a higher suspicion of babesiosis or malaria. However, due to limited travel history, malaria was ruled out. He further had testing for Anaplasma PCR and Lyme's, which had not returned before the patient was discharged. 

Treatment

Our investigations in patient #1 were diagnostic of tick-borne illnesses. So he was empirically treated for babesiosis, Anaplasma, and Lyme's diseases.

The patient was treated with atovaquone 750 mg every 12 hours, azithromycin 250 mg daily, and doxycycline 100 mg twice daily for a total duration of 10 days. He also received fluids during the hospitalization, which improved/resolved the acute kidney injury (AKI).

Outcome and Follow-Up

The patient continued to improve over the course of treatment. His sodium normalized, creatinine normalized, and liver function tests (LFTs) trended down on discharge. Lactate dehydrogenase (LDH) has continued to trend down and hemoglobin has trended down to 8.5 but further improved to 9.1 upon discharge.

The patient followed up with an infectious diseases specialist one week after discharge. Labs obtained at the time had an ALT and AST of 32 and 36. CBC showed hemoglobin and hematocrit (H&H) of 10.4 and 32.9, a normal platelet level of 343, and a normal WBC count of 6.4. The parasitic burden was 0.0x2. Table 1 shows a comparison of labs on presentation and on follow-up after discharge for our first patient along with reference ranges for the corresponding values (Table 1).

**Table 1:** Labs of our first patient ALT: alanine transaminase; AST: aspartate aminotransferase; LDH: lactate dehydrogenase

CMP	On Presentation	On Follow-up	Reference Ranges
Sodium (mmol/L)	132	139	134-144
Creatinine (mg/dL)	1.57 – unknown baseline	1.06	0.7 – 1.2
Total bilirubin (mg/dL)	2.1	0.4	0.0-1.2
Direct bilirubin (mg/dL)	1.1	NA	0.1-0.5
ALT (IU/L)	127	32	0 – 55
AST (IU/L)	210	36	5 – 34
CBC			
Hemoglobin (gm/dL)	12.7	10.4	11.1 – 15.9
Hematocrit (%)	37.3	32.9	34.0 – 46.6
Red cell distribution width (RDW) (%)	16.7	20.8	11.7-15.4
Mean corpuscular volume (MCV) (fl)	80.7	88	79-97
Platelets (10^3^mcL)	43	343	150 – 450
Reticulocyte count (%)	2.3	NA	0.4-2.0
Immature reticulocyte fraction (IRF) (%)	16.7	NA	2.3-13.4
Parasitic load on peripheral smear (%)	1.8	0	0
LDH (IU/L)	752	NA	125 - 220
Haptoglobin (mg/dL)	8.0	NA	14 - 258

Case #2

This was a 71-year-old male who presented to the hospital with fatigue, lightheadedness, ataxia, confusion, dyspnea on exertion, night sweats, fevers, urinary frequency, and urgency. He has a past medical history of chronic obstructive pulmonary disease (COPD), hypertension, hyperlipidemia, cervical radiculopathy, degenerative disc disease with spondylolisthesis at lumbar spines levels 4 and 5 (L4-L5), and insomnia. He had a T-max of 103.5 Fahrenheit at home, which prompted him to visit urgent care, where he was diagnosed with a sinus infection and was started on cefdinir 300 mg twice daily. His fever curve has initially improved. However, his wife noticed him to be more febrile, associated with severe confusion, generalized weakness, and gait instability. He was further brought to the hospital because of these manifestations. He had a recent trip to Maryland where he first noticed fevers, but he had no history of travel outside of the country.

On presentation to the hospital, he had significant hypotension, symptomatic anemia, and tachycardia. On physical exam, the notable finding was a systolic murmur in the left lower sternal border.

He was nonreactive to volume resuscitation and blood transfusion, and he was required to transfer to the intensive care unit (ICU), and he required pressors for a brief period.

Investigations

On admission, his investigations had a sodium level of 131, ALT and AST of 50 and 63, respectively, H&H of 7.8 and 21.2, and low platelets of 60. His peripheral smear showed parasitemia, with a parasitic load of 1.5. He also had a high LDH and low haptoglobin of 805 and 8.0, respectively.

Urinalysis (UA) was negative, Coombs and HIV were negative, and procalcitonin was low at 0.35. 

Antibodies for Mycoplasma, Anaplasma, Lyme's, and Babesia were sent. These did not result during the hospitalization. His Anaplasma antibodies came back negative, Babesia IgG was positive, Lyme's was positive, and Mycoplasma was positive.

CT head was negative for acute hemorrhage, mass effect, or infarction.

Peripheral Smear

**Figure 2:**
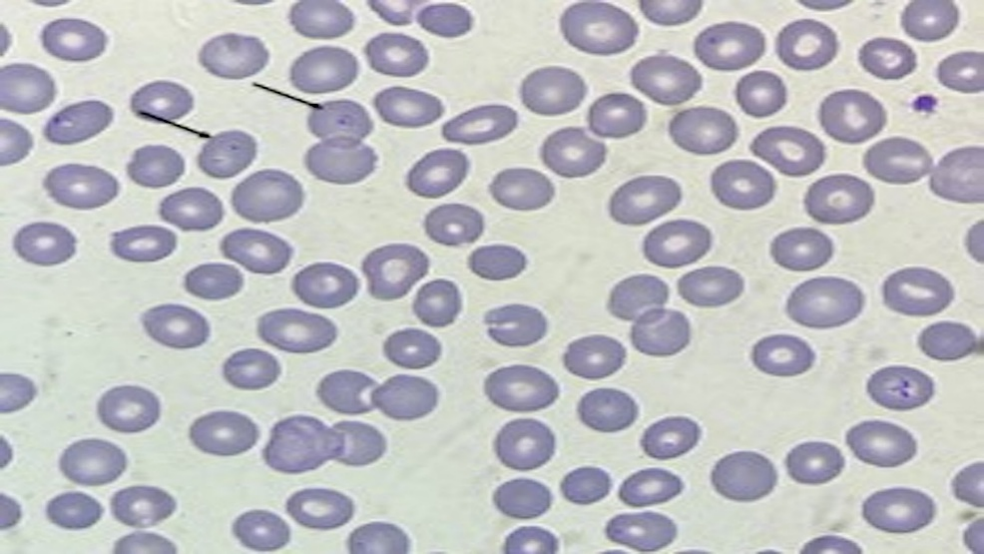
With arrows toward the intracellular ring-like parasites from the patient in Case #2

Peripheral smear also had intracellular ring-like parasites on examination (Figure 2).

Differential Diagnosis

Differential diagnoses included babesiosis, Mycoplasma, Lyme's disease, and sepsis secondary to unknown sources in patient #2. Peripheral smear showed parasites and ringed erythrocytes as in both patient #1 and patient #2, which made babesiosis more likely. His blood cultures were negative, which ruled out sepsis. We further tested the patient for antibodies for babesiosis, Mycoplasma, and Lyme's, which came back positive for all the above after the patient was discharged from the hospital. Multiple tickborne infections/coinfections with other tickborne diseases are uncommon, however, they do occur. Mycoplasma is a possible cause of hemolytic anemia in this patient. However, it can also be due to babesiosis.

Treatment

Due to hemolytic anemia and the Coombs test being negative, patient #2 was treated for babesiosis, Lyme's, and Mycoplasma.

The patient was initially started on broad-spectrum antibiotics, vancomycin and piperacillin-tazobactam, for suspicion of sepsis, further streamlined to azithromycin 500 mg daily followed by 250 mg, atovaquone 750 mg twice daily for babesiosis and doxycycline 100 mg every 12 hours to cover for Lyme's disease to finish a total course of 10 days of antibiotics. He also received a blood transfusion along with fluid resuscitation and maintenance fluids. He also required pressor support along with midodrine for hypotension.

Outcome and Follow-Up

His lab values and symptoms greatly improved following treatment. He was discharged eight days after admission, and his lab values were as follows:

CBC had an H&H of 9.2 and 26.1, platelets stayed low at 46, and the peripheral smear was still positive for parasites at the time of discharge.

The patient presented to the hospital a month later from discharge with substernal chest pain that woke him up from sleep. During this visit, he had a negative workup with troponins, electrocardiogram (EKG), and CT with the pulmonary embolus protocol (CT PE). His chest pain has improved but he continued to have mild pleuritic chest pain. He was further discharged after the negative evaluations and was asked to follow up with cardiology to have a full ischemic workup.

Labs at this later presentation showed normal sodium, CBC had a WBC of 10.9, H&H of 11.8 and 36.1, which were significantly improved, and platelets normalized to 267. He had no parasitemia during this presentation. Table 2 shows a comparison of labs on presentation and further on follow-up after discharge for our second patient along with normal values corresponding to the labs (Table 2).

**Table 2:** Labs for our second patient ALT: alanine transaminase; AST: aspartate aminotransferase; LDH: lactate dehydrogenase

CMP	On Presentation	On Follow-up	Reference Ranges
Sodium (mmol/L)	131	139	134-144
Creatinine (mg/dL)	1.06	0.65	0.7 – 1.2
Total bilirubin (mg/dL)	1.6	NA	0.0-1.2
Direct bilirubin (mg/dL)	0.8	NA	0.1-0.5
ALT (IU/L)	50	32	0 – 55
AST (IU/L)	63	36	5 – 34
CBC			
Hemoglobin (gm/dL)	7.8	11.8	11.1 – 15.9
Hematocrit (%)	21.2	36.1	34.0 – 46.6
Red cell distribution width (RDW) (%)	15.1	15.2	11.7-15.4
Mean corpuscular volume (MCV) (fl)	82.5	99.4	79-97
Platelets (10^3^mcL)	60	267	150 – 450
Reticulocyte count (%)	2.0	NA	0.4-2.0
Immature reticulocyte fraction (IRF) (%)	7.2	NA	2.3-13.4
Parasitic load on peripheral smear (%)	1.5	0	0
LDH (IU/L)	805	NA	125 - 220
Haptoglobin (mg/dL)	8.0	NA	14 - 258

## Discussion

Babesiosis is an infectious disease, and the most common culprit is a protozoan Babesia microtia (B. microtia) in the United States, especially in the Northeastern United States. It is a tick-borne disease caused primarily by Ixodes scapularis. A rare mode of transmission is through contaminated blood products. There were two cases reported to the Centers for Disease Control (CDC) in 2018 [7].

Babesiosis was known to mankind as early as 1888. It was first identified as intra-erythrocytic microorganisms in cattle. This disease derives its name from Victor Babes, the pathologist and the microbiologist who identified it. The first reported or documented case of babesiosis in the human population is from a paper published in 1957. This was in a 33-year-old male with a prior history of splenectomy from an abdominal trauma, who rapidly deteriorated and expired within less than seven days of symptom onset [8]. The first documented case of the immunocompetent patient was in 1969 in the state of Massachusetts, Nantucket Island [9].

The estimated incidence of babesiosis is 2000 cases yearly, but it is thought to be grossly underreported, and the incidence is suspected to be much higher and increasing [10]. The incubation period of B. microti infection following a tick bite is one to four weeks. The incubation period after transfusion of contaminated blood products is usually three to seven weeks but can range from one week to six months [1-2,11]. The severity of the infection can be very varied ranging from asymptomatic infection to severely fatal infection. This depends on the patient's immune status. Most common symptoms include fever, fatigue and malaise, chills and sweats, headache, anorexia, arthralgia, and nausea. Rarely, they can be accompanied by vomiting, sore throat, abdominal pain, conjunctival injection, photophobia, weight loss, emotional lability, depression, and hyperesthesia [3-6,12-13].

Diagnosing babesiosis is done with peripheral blood smear examination or PCR. Antibody testing for Babesia is not effective for the diagnosis, as it can be positive even after a year after infection. Even if antibody testing is done, it should be followed up with either peripheral blood smear examination or PCR.

Treatment of babesiosis is primarily with atovaquone plus azithromycin or a combination of clindamycin plus quinine. The duration of treatment for immunocompetent patients is about seven to 10 days (about one and a half weeks). The dosage required for treatment in severe diseases requiring hospitalization is atovaquone 750 mg orally every 12 hours plus azithromycin 500 mg intravenous (IV) every 24 hours until symptoms abate and then transition to atovaquone 750 mg orally every 12 hours plus azithromycin 250 to 500 mg orally every 24 hours to complete a seven to 10-day course in total. Alternative treatment is clindamycin 600 mg IV every six hours plus quinine sulfate 542 mg base (which equals 650 mg salt) orally every six to eight hours and then transition all of them to oral dosing to complete a total course of seven to 10 days.

In highly immunocompromised patients, however, the duration of treatment is prolonged antibiotics for about six consecutive weeks. The treatment of choice is still atovaquone plus azithromycin, dosage with atovaquone 750 mg orally every 12 hours plus azithromycin 500 mg IV every 24 hours and then transition to oral azithromycin as symptoms abate, but a 500-1000 mg daily dose of oral azithromycin is recommended. Alternative treatment is clindamycin 600 mg IV every six hours plus quinine sulfate 650 mg orally every eight hours until symptoms abate and then convert all of them to oral therapy. This is also for a total duration of seven to 10 days (Table 3).

**Table 3:** Treatment of babesiosis

Treatment	Preferred	Alternative
Babesiosis in Immunocompetent hospitalized patients	Atovaquone 750 mg orally every 12 hours plus azithromycin 500 mg intravenous (IV) every 24 hours until symptoms abate, then transition to atovaquone 750 mg orally every 12 hours plus azithromycin 250 to 500 mg orally every 24 hours to complete a 7 to 10-day course in total	Clindamycin 600 mg IV every 6 hours plus quinine sulfate 542 mg base (which equals 650 mg salt) orally every 6 to 8 hours, then transition all of them to oral dosing to complete a total course of 7 to 10 days.
Babesiosis in Immunocompromised patients	Atovaquone 750 mg orally every 12 hours plus azithromycin 500 mg IV every 24 hours, then transition to oral azithromycin as symptoms abate, but the dosage of oral azithromycin of 500 -1000 mg daily dose is recommended. The total duration of treatment is 6 consecutive weeks.	Alternative treatment is clindamycin 600 mg IV every 6 hours plus quinine sulfate 650 mg orally every 8 hours until symptoms abate, then convert all of them to oral therapy. This is also for a total duration of 6 consecutive weeks.

In patients with high parasitemia, exchange transfusion using red blood cells can be used, however, data is quite limited on the efficacy of this modality of treatment.

This is one of the diseases where infectious disease consultation is quite warranted and helpful. Follow-up after hospital discharge is vital to monitor for Babesia parasitemia using peripheral blood smears, especially in immunocompromised patients. If the acute illness persists, guidelines recommend monitoring with peripheral blood smears for Babesia parasitemia. After the symptoms are resolved, this can be stopped in immunocompetent patients [14].

During the literature review, we have found that neurologic manifestations associated with babesiosis were exceedingly rare, cognitive dysfunction and motor dysfunction being the rarest, and pathogenesis for this is unclear [15-16].

We have found very few cases with neurologic manifestations with tick-borne infections, most of them with Lyme's disease and anaplasmosis rather than with babesiosis. There were reported cases of neurologic manifestations in animal populations, not so much in human populations. There is a huge gap in knowledge about tick-borne infections, especially babesiosis, which is grossly under-studied and underdiagnosed [17]. As the incidence of tick-borne diseases is increasing, especially babesiosis, it poses a need for further study and exploration of the disease. This is a diagnosis associated with complete recovery on prompt diagnosis and treatment, and it could be fatal if left undiagnosed or with a delayed diagnosis.

## Conclusions

Although the patient presented with stroke-like/transient ischemic attack (TIA)-like signs and symptoms, it is essential to get a good history in order to rule in or rule out differential diagnoses. Once we narrow down the possible differential diagnosis, it is crucial to arrive at a confirmatory diagnosis. Even though neurologic manifestations are rare with babesiosis, unlike other tickborne diseases, it is pertinent to have a high suspicion for this disease, especially in an endemic area such as the Northeast United States. It is important to have a high suspicion of this disease when neurologic manifestations alongside hematologic manifestations are seen. Early diagnosis and treatment are detrimental to recovery; delayed diagnosis could be fatal.
